# A machine learning model for predicting six-month rehospitalization in heart failure patients with multiple comorbidities

**DOI:** 10.3389/fcvm.2026.1612592

**Published:** 2026-09-18

**Authors:** Rahul Awasthy, Michael L. Seavers, Aayush Awasthy

**Affiliations:** Data Science, Harrisburg University of Science and Technology, Harrisburg, PA, United States

**Keywords:** artificial intelligence, HCUP, heart failure, machine learning, physionet, prioritization

## Abstract

This research (retrospective cohort study) investigates how tailored discharge instructions for heart failure (HF) patients with multiple health conditions can lower their emergency or urgent care readmission risk. Utilizing an AI/ML approach, this study develops a model that classifies HF patients' readmission risk at discharge by evaluating approximately 20 parameters across five categories, including comorbidities (e.g., liver disease, diabetes), patient demographics (e.g., age, BMI), clinical data (e.g., lipid and blood levels), diagnostic parameters (e.g., ejection fraction, HF type), discharge details (e.g., discharge destination and location), and medication history. The AI model identifies high-risk patients, who are then prescribed a more focused wellness and rehabilitation plan, including in-person visits and regular monitoring. Lower-risk patients are given less rigorous plans, involving virtual check-ins and self-management tools. In contrast, high-risk patients can undergo intensive wellness plans, involving hospital/clinical visits and tracking by wellness staff. The study's approach of customizing post-discharge care based on re-admission risk aims to reduce re-admissions and improve HF patient outcomes. This research employs key ML models on the PhysioNet dataset, a collection of hospital admissions and mortality records for HF patients from Zigong Fourth People's Hospital in Sichuan, China (2016-2019). To evaluate the broader applicability of the developed models, their results on the PhysioNet dataset are benchmarked against the HCUP Maryland SID (2014), a secondary dataset of HF patients. The ML models exhibited strong performance, achieving efficiencies exceeding 76% and specificities reaching 97% in identifying HF patients with a higher risk of readmission and ranking them according to their risk level. The study's approach to customizing post-discharge care based on readmission risk aims to reduce readmissions and improve HF patient outcomes. Thus, this presents a comprehensive and reliable strategy for HF management and wellness.

## 1 Introduction

### 1.1 Background

Based on their physical condition, clinical history, demographics, biochemical data, and diagnosis, HF patients' correct identification and prioritization are vital for assigning them to the most suitable rehabilitation (Scalvini et al., European Journal of Preventive Cardiology, 2019) (1) or wellness program. This research introduces an automated machine learning model that can effectively help assign suitable rehabilitation programs for patients with HF and comorbidities when utilized by healthcare professionals. With its potential to substantially decrease readmissions and avert high-risk emergencies, this model offers a pathway to improved patient outcomes and a less burdened healthcare system (Upadhyay et al., Inquiry, 2019) (2).

### 1.2 Importance

HF patients with multiple comorbidities require a prioritization method not just at the time of healthcare facility admissions but also at the time of discharge (3). Currently, rehabilitation and wellness are not established paths for HF patients. This research studies how providing proper guidance at the time of release to critical or high-risk HF patients can significantly reduce the chances of their re-admission to the emergency room. Discharge guidance should refer to a patient-centric AI/ML model patient classification (as a decision support tool) during HF patient discharge to reduce patients' re-admission chances post-discharge (4). This ML model would rank HF patients from high to low risk based on the re-admission risk of HF patients.

An automated machine learning model takes patient health conditions as input at discharge and identifies their chances of getting readmitted. Patients with higher chances of re-admission (5) are prescribed a more rigorous wellness/rehabilitation path that may include personal visits, regular checking with diagnostic parameters, and using tools and techniques for daily health updates. Patients with medium to lower risks see less rigor (maybe more relaxed) in tracking health conditions by using a combination of virtual appointments/check-ins, checking diagnostic parameters less frequently, and practical usage of mobile/online applications to allow patients to improve with self-management. Implementing this AI-based automated patient classification/allocation of wellness path reduces the chances of HF patients with comorbidities getting readmitted under emergency/critical conditions.

### 1.3 Goals of investigation

This study has two primary objectives: (1) to design and implement a data collection architecture capable of capturing clinical and non-clinical variables for HF patients at the time of discharge, and (2) to use these collected variables to develop and evaluate a machine learning model that predicts the risk of hospital rehospitalization within six months of discharge, thereby supporting the assignment of patients to an appropriate wellness/rehabilitation pathway. To this end, the overall architecture or process flow is divided into three tiers. Tier 1 collects clinical and non-clinical data for HF patients in hospital or telemedicine cases (6). Once the data is collected through wearable devices, blood tests, diagnostic biochemical (electrolytes), or clinical parameters in hospital/home/path labs, health staff/patient representatives can enter data into mobile apps/web pages. Tier 2 loads all the patient data in databases on premise or in the cloud to pass it further to Tier 3, in which referral classification and prioritization models identify HF patients' wellness decision paths.

Based on clinical risk factors, the generated classification (in Tier 3) of HF patients is used to apply the right wellness decision paths for each classified group/bucket. The suggested wellness path for Tier 3 is (1) High-Risk Patients (Need personal visits, eHealth cloud applications, mobile app/follow-up, and doctor's regular touchpoints) (2) Medium Risk Patients (Self-management application and less regular Doctors/Wellness coach visits) (3) Lower Risk Patients (Self-Management Application and limited Doctors/Wellness coach help/interventions).

## 2 Materials and methods

### 2.1 Study design and setting

This is a retrospective, multi-center cohort study using de-identified secondary data from two existing hospital administrative/clinical databases (PhysioNet, from Zigong Fourth People's Hospital, Sichuan, China, 2016–2019; and HCUP Maryland State Inpatient Database, 2014), described further in the Participants and Data Sources subsection below. This project generates an automated machine learning model. It creates the wellness/rehabilitation decision path (as a decision support tool for wellness health staff) for HF patients based on patient criticality, age, the possibility of mortality, re-admissions to the hospital, comorbidities, and availability of health insurance. The rehabilitation decision path is based on research completed in the past to reduce the re-admission of HF patients with multiple comorbidities. The referral mechanism suggested in the study will help HF patients handle their secondary problems, such as heart problems, and manage other comorbidities along with the primary illness. With this, complete care and wellness could be planned for the HF patient. Once the patient is classified into high, medium, and low-risk patients, a patient ranking is generated for each classification bucket to help the wellness/rehabilitation team give special care to patients with higher risk. The Institutional Review Board at Harrisburg University of Science and Technology determined this research was not subject to ethical review (Waived Ethical Review number: IRB 20231029). This study is reported in accordance with the Strengthening the Reporting of Observational Studies in Epidemiology (STROBE) Statement for cohort studies; a completed STROBE checklist indicating where each item is addressed in the manuscript is provided as a supplementary file.

### 2.2 Participants and data sources

The primary dataset, PhysioNet, aims to facilitate epidemiological studies of HF and is vital in providing optimal care to reduce patient populations and healthcare system differences between different countries (7, 8). The dataset includes 168 variables for 2,008 patients with HF (Zhang, Z. et al., 8), of which overall 44 key parameters are identified for HF patients to determine the chances of hospital re-admission after initial hospitalization (9). Twenty-five numerical and 19 categorical data variables for HF patients were shortlisted to identify the HF patients' re-admission events (refer to Tables 1, 2). Subsequent hospital admissions and mortality outcomes were ascertained through mandatory follow-up assessments conducted at 28 days, three months, and six months post-discharge. The database also encompasses records of medications administered during the index hospitalization, with diuretics (furosemide, torsemide, spironolactone), inotropes (deslanoside, dobutamine, digoxin, isoprenaline, milrinone), and vasodilators (10) representing the primary pharmacological categories. The vasodilator drug had isosorbide mononitrate and nitroglycerin (11).

**Table 1:** Dependent variable—re-admission.

Variable	Description	Data type
Visit to the emergency department within six months	HF patient death within six months	Binary

The above Table shows the dependent variable used in the re-admission Prioritization model.

**Table 2:** Independent variables—re-admission.

Variables	Description	Data type
Inpatient number	The unique patient ID	Continuous
Destination Discharge	The destination of hospital discharge, recorded after discharge	Continuous
Admission ward	First admission ward	Continuous
Admission way	Possible ways of admission are Emergency vs. non-emergency	Binary
Discharge department	The patient was discharged from	Continuous
Gender	Gender: Male, Female	Binary
Body temperature	Body temperature in degrees Celsius	Continuous
Pulse	Pulse rate (beats per minute)	Continuous
Respiration	Respiratory rate (breaths/min)	Continuous
Systolic blood pressure	Systolic blood pressure (mmHg)	Continuous
Diastolic blood pressure	Diastolic blood pressure (mmHg)	Continuous
BMI	BMI (kg/m^2^)	Continuous
Type of HF	Type of HF (left, right, both)	Continuous
Myocardial infarction	Myocardial infarction	Binary
Congestive HF	Congestive HF (HF for the first time in	Binary
	This is an index hospitalization.)	
Dementia	Dementia	Binary
Chronic obstructive pulmonary disease	Chronic obstructive pulmonary disease	Binary
Peptic ulcer disease	Peptic ulcer disease	Binary
Diabetes	Diabetes	Binary
Moderate to severe chronic kidney disease	Moderate to severe chronic kidney disease with	Binary
	Glomerular filtration rate <60 mL/min	
Solid tumor	Solid tumor	Binary
Liver disease	Liver disease	Binary
AIDS	AIDS	Binary
CCI score	Charlson Comorbidity Index score	Continuous
Type II respiratory failure	Type II respiratory failure	Binary
fio2	Fraction of inspired oxygenation (%)	Continuous
Acute renal failure	Presence of acute kidney injury	Binary
Outcome during hospitalization	Outcome during hospitalization, recorded after the decision of discharge (living vs. dying in the hospital)	Continuous
Return to the emergency.	Patients return to the emergency department within six months	Binary
department within six months		
Time to the emergency	Patient time to the emergency department within six months	Continuous
department within six months		
Calcium	Calcium (mmol/L); ref: 2.11–2.52	Continuous
Potassium	Potassium (mmol/L); ref:3.5–5.3	Continuous
Chloride	Chloride (mmol/L); ref:99–110	Continuous
Sodium	Sodium (mmol/L); ref:137–147	Continuous
Inorganic Phosphorus	Inorganic Phosphorus (mmol/L); ref: 0.85–1.51	Continuous
Serum magnesium	Serum magnesium (mmol/L); ref:0.75–1.02	Continuous
Creatine kinase isoenzyme	Creatine kinase isoenzyme to creatine kinase; ref: 0–0.05	Continuous
to creatine kinase		
Creatine kinase	Creatine kinase (IU/L); ref: 22–270	Continuous
Total protein	Total protein (g/L); ref: 65–85	Continuous
Cholesterol	Cholesterol (mmol/L); ref: 2.9–5.68	Continuous
Low-density lipoprotein	Low density lipoprotein Cholesterol (mmol/L); ref: 0–3.36	Continuous
cholesterol		
Triglyceride	Triglyceride (mmol/L); ref:0.22–2.26	Continuous
High-density lipoprotein	High density lipoprotein cholesterol (mmol/L); ref:0.9–2.19	Continuous
cholesterol		
Blood glucose (A1C)	Blood glucose (mmol/L); ref: 3.9–6.1	Continuous
Lactate	Lactate (mmol/L); ref: 0.5–2.2	Continuous
Age Cat	Patient age categorized in decades	Continuous

The above Table shows the set of independent variables used in the readmission Prioritization model.

The research further verifies the results of patient prioritization (12) outcomes from the PhysioNet dataset with the secondary dataset HCUP MD SID for 2014, with HF patients, to verify the applicability of results in more comprehensive healthcare settings across geographies and different patient settings. The SID database holds approximately 48,000 records of HF admissions to Maryland hospitals in 2014.

### 2.3 Eligibility criteria and study size

Inclusion and exclusion criteria were applied separately to each dataset and then harmonized to a common analytic definition. For the primary PhysioNet cohort, eligible records were consecutive adult patients (aged 18 years or older) admitted to Zigong Fourth People's Hospital between December 2016 and June 2019 with a confirmed diagnosis of HF recorded as an ICD-9 code and meeting the 2016 European Society of Cardiology (ESC) diagnostic criteria for HF. The index admission was defined as the first HF hospitalization captured in the record for a given patient during the study window. Records were excluded if the HF diagnosis could not be confirmed against the ESC criteria, if the patient died during the index hospitalization (an in-hospital death is a competing event that precludes six-month readmission and would otherwise bias the outcome), or if the six-month follow-up status could not be ascertained through the mandatory 28-day, three-month, and six-month follow-up assessments. Of the 2,008 HF patients available in the source dataset, records with an unconfirmed HF diagnosis, in-hospital death, or incomplete six-month follow-up were removed; the resulting analytic cohort was used for model development and evaluation, and missing values in individual predictor fields were handled by K-nearest-neighbor imputation rather than case-wise deletion so that the analytic sample size was preserved. For the secondary HCUP Maryland State Inpatient Database (SID) 2014 cohort, eligible records were inpatient hospital discharges from Maryland acute-care hospitals in calendar year 2014 with a principal or secondary diagnosis of HF identified by the corresponding ICD-9-CM diagnosis codes; non-heart-failure admissions, observation-only stays, and records lacking the fields required for the five harmonized variable categories were excluded, yielding approximately 48,000 HF hospital admission records used for cross-dataset verification. Because the two datasets are administrative databases without a shared patient identifier, the HCUP cohort was used to verify the transferability of the variable set and model behavior rather than to pool patient-level records with PhysioNet. The PhysioNet dataset comprises 2,008 HF patients (168 variables), and the HCUP Maryland SID 2014 secondary dataset comprises approximately 48,000 HF hospital admission records, as described above.

### 2.4 Variables

The predictor variables considered for the classification and prioritization models fall into five categories, consistent with the exploratory analysis presented in the Results: (1) comorbidities (chronic and non-chronic, e.g., CKD, diabetes, COPD), (2) patient type (age, BMI, gender), (3) clinical/biochemical data (e.g., HDL, LDL, calcium, potassium, triglycerides), (4) diagnostic parameters (e.g., LVEF, mitral valve opening values), and (5) discharge place/destination. The primary outcome variable is hospital rehospitalization within six months of discharge, ascertained through mandatory follow-up assessments at 28 days, three months, and six months post-discharge. To clarify the timing of measurement, all baseline clinical characteristics (body temperature, pulse, respiration rate, systolic and diastolic blood pressure, mean arterial pressure, weight, height, BMI, NYHA class, Killip grade, and Glasgow Coma Scale score), laboratory findings, and comorbidities were recorded on day one of the index hospital admission rather than at discharge; demographic and discharge-related variables (discharge department and discharge destination) were recorded at the time of discharge. This distinction is clinically important because the interpretation of laboratory values, body weight, and blood pressure differs substantially between admission and discharge, and it is stated here to avoid ambiguity in how the predictor variables should be interpreted. With respect to the outcome, the PhysioNet source data record rehospitalization as an all-cause event and do not natively provide a validated HF-specific readmission flag; accordingly, the primary outcome in this study is all-cause rehospitalization within six months. This is a recognized limitation because non-cardiovascular admissions are also elevated in older, multimorbid, and frail patients, and it is discussed further in the Limitations section.

### 2.5 Data collection procedure and statistical methods

The real-time patient monitoring and prioritization model generated in this research (Figure 1) utilizes various clinical and non-clinical inputs for HF patients, sourced manually or through wearable devices/user interfaces.

**Figure 1:**
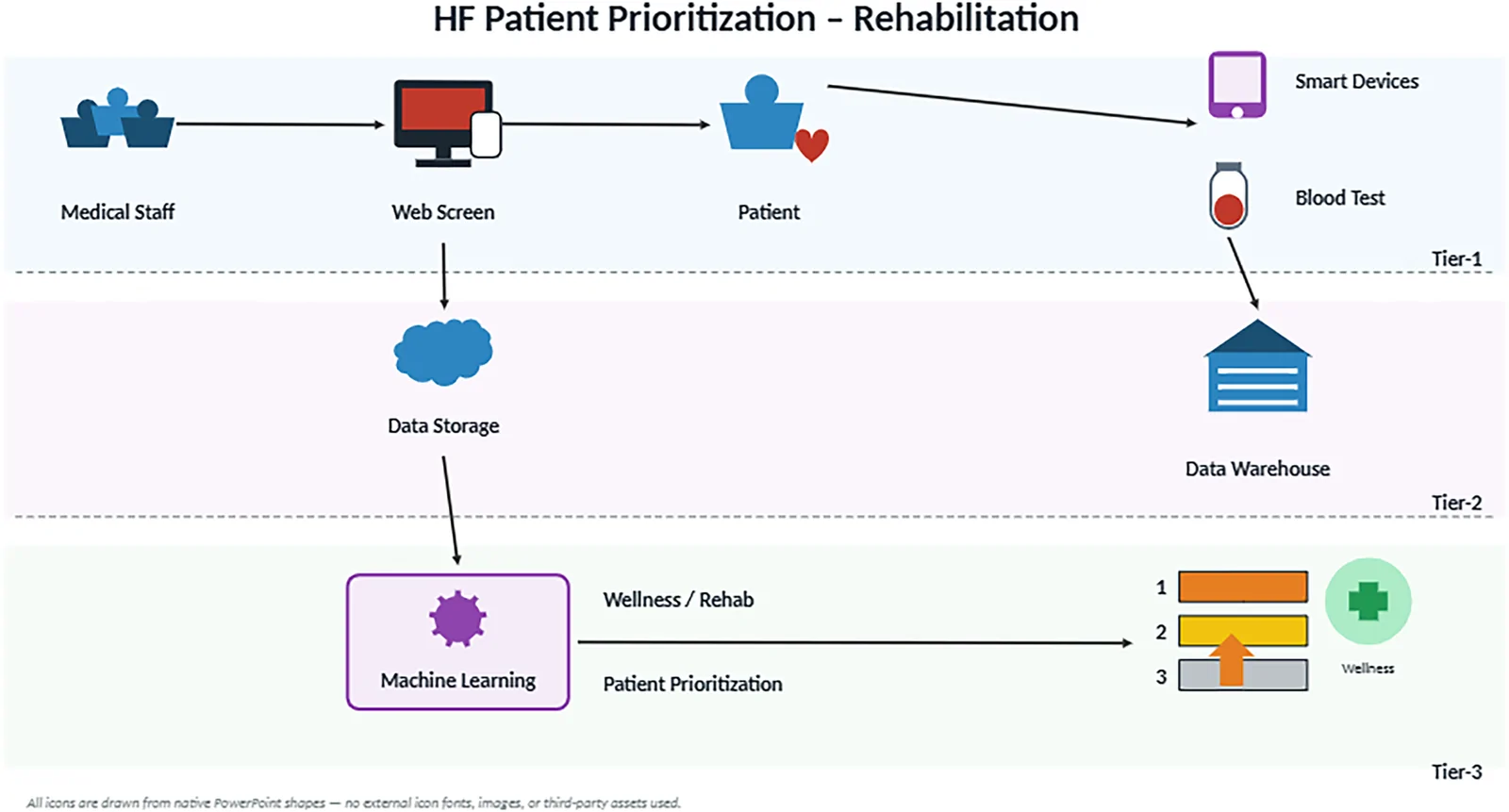
Three-tiered prioritization approach for HF patients during the discharge process. The figure has been regenerated so that it contains only the schematic workflow (Tier 1 data collection, Tier 2 data storage, and Tier 3 machine learning and wellness-pathway assignment); any editing-software icon inadvertently retained from figure preparation has been removed and carries no analytic meaning.

#### 2.5.1 Clinical and/or observational parameters (Tier 1)

The user interface collects clinical and non-clinical data on HF patients in an inpatient hospital setting or during telemedicine consultations. Once the data is collected through wearable devices, blood tests, diagnostic biochemical (electrolytes), or clinical parameters in hospital/home/path labs, health staff, patients, or representatives enter it into mobile apps or web pages.

#### 2.5.2 Online data collection systems (Tier 2)

Online data collection utilizes a range of systems, such as mobile phones, laptops, and clinical data collection machines, to send clinical data to server-side machines for real-time machine learning model execution. These collection systems include transferring captured HF patient data to a data store, server, or cloud.

#### 2.5.3 Real-time machine learning models (Tier 3)

Tier 3 of this research centers on developing a machine learning model for HF patient classification and prioritization. The following steps are undertaken:

- **Data Imputation:** Missing data is addressed using K-nearest neighbor (KNN) imputation, an analytical technique that replaces missing values to preserve dataset information and offers a robust estimation method.
- **Classification and Prioritization:** Machine learning models such as Support Vector Machine (SVM), logistic regression, random forest, and linear regression are employed to classify HF patients into risk buckets/groups and prioritize them by ranking them from high to low risk. The model with the optimal outcome will be chosen for the final version.
- **Causal Inference Modeling:** A mathematical model for causal inference (linear regression, Bayesian, or Binomial) is created to account for multiple causal factors. This analysis aims to determine the impact of various admission-related independent variables on HF patient mortality and compare their relative influence.
- **Critical HF Patient Ranking:** HF patients are ranked according to their risk level, from high to low, based on their predicted probability of hospital readmission within the subsequent six months.
- **Verification**: In this step, the functioning of the HF patient prioritization readmission model generated with the PhysioNet dataset is verified by running the models on the HF dataset from patient hospitalization for HCUP Maryland state 2014 to ascertain the uniformity of model results across the board (13).
- **Validation**: The predictive model's results identify all independent variables' regression coefficients/weights. These weights or risk factors are compared to past research work for the Canadian Triage and Acuity Scale (CTAS) to verify how the current model performs compared to the past patient prioritization models (14).

### 2.6 Bias

Several potential sources of bias were considered and addressed. Selection bias is a concern because the study draws on two administrative datasets from different health systems, countries, and time periods: the single-center PhysioNet cohort from Zigong Fourth People's Hospital in China (2016–2019) and the state-wide HCUP Maryland SID in the United States (2014). To limit its impact, the two datasets were not pooled at the patient level; instead, PhysioNet served as the development cohort and HCUP was used only to verify that the same variable categories and model behavior transfer to an independent population, so that any center- or country-specific structure in one dataset could not silently drive the reported model. Restriction was applied by including only confirmed HF admissions in both cohorts and by defining a consistent index admission, which reduces spectrum bias from ambiguous or non-heart-failure cases. Information and measurement bias is a further concern because clinical values can shift over the course of a hospitalization; to mitigate this, all baseline clinical characteristics, laboratory findings, and comorbidities in the PhysioNet cohort were fixed at a single, consistent timepoint (day one of the index admission), while discharge-related variables were taken at discharge, so that predictors are interpreted at a uniform point in the patient journey (see the timing clarification in the Variables section and the reviewer comment on Line 87). Missing-data bias was addressed with K-nearest-neighbor imputation rather than complete-case deletion, preserving the analytic sample and reducing bias from non-random dropout of individual fields, and the sensitivity of the models to variable count was examined by re-fitting on 44, 22, and 7 variable subsets. Residual confounding and the absence of an adjudicated cause-of-admission field (which limits the outcome to all-cause rather than heart-failure-specific readmission) remain, and are disclosed in the Limitations section.

## 3 Results

### 3.1 Participants

Participant flow is reported separately for the two cohorts. For the primary PhysioNet cohort, 2,008 HF patients were initially available in the source database. Patients were removed at successive stages for an unconfirmed HF diagnosis when checked against the ESC criteria, for in-hospital death during the index admission (a competing event that precludes a six-month readmission outcome), and for missing six-month follow-up status; the remaining patients formed the analytic cohort used for model development, ranking, and evaluation. Rather than excluding patients with isolated missing predictor values, missing entries in individual fields (for example, ECG-derived measures, oxygen saturation, peptic ulcer status, chronic kidney disease, left ventricular ejection fraction, and the Charlson Comorbidity Index) were completed by K-nearest-neighbor imputation, so that the analytic sample size was preserved rather than reduced by list-wise deletion. The analytic cohort was then randomly partitioned into training (80%) and test (20%) subsets, with model fitting and hyperparameter tuning performed only on the training subset and performance reported on the held-out test subset. For the secondary HCUP Maryland SID 2014 cohort, all 2014 Maryland inpatient discharges were screened, records without a HF diagnosis code and those lacking the fields needed for the five harmonized variable categories were excluded, and approximately 48,000 HF hospital admission records were retained for cross-dataset verification. Because HCUP was used to verify variable transferability rather than to train the model, no patient-level train/test split was applied to that cohort. A participant flow diagram summarizing records available, records excluded with reasons, and the final analytic sample for each dataset can accompany this section in the supplementary material.

### 3.2 Descriptive data: baseline characteristics (data imputation)

The KNN data imputation technique fills in missing data for the ECG-EMS, ECG-AMS, Oxygen Saturation, Peptic Ulcer, chronic kidney disease (CKD), Left ventricular ejection fraction (LVEF), and Charlson Comorbidity Index (CCI) score columns. The input data is then divided into five key segments to compare patients readmitted within six months of their initial (most recent) hospital stay with those who were not. These five areas to classify the input data are Comorbidities (chronic and non-chronic), Patient Type, Patient Clinical/Biochemical data, Patient Diagnostic parameters, and Discharge Place after HF patients complete their current hospital services before getting discharged from the inpatient facility.

### 3.3 Descriptive data: exploratory analysis by variable category (outcome-stratified)

#### 3.3.1 First classification

The first bucket is Comorbidities, which identifies different comorbidities in HF patients' data. The HF patient data predominantly featured comorbidities such as liver disease, CKD, renal failure, diabetes, dementia, and chronic obstructive pulmonary disease (COPD). CKD and Diabetes are the two comorbidities found in most HF patients who were readmitted in 6 months (15) (refer to Supplementary Table S1).

#### 3.3.2 Second classification

Another set of variables that need to be understood to reduce the re-admission of HF patients is Patient Type, which mainly includes their Age, BMI, and gender details. It is identified that older HF patients (older than 60 years) have higher chances of re-admission (16), and as the age increases across successive decade bands, the chances of re-admission increase as well. For consistency, age was analyzed by decade throughout the study, and Supplementary Table S2 reports age using the same decade-based categorization applied in Table 2 rather than a single broad band. The female population is more prone to re-admission than male HF patients (refer to Supplementary Table S2).

#### 3.3.3 Third classification

The Next bucket of key variables is Clinical/Biochemical data. Once the HF patient has been admitted to any hospital or facility, it is easier to get the Biochemical parameters using the blood sample. High-density lipoprotein (HDL), Calcium, and Potassium content showed some connection for readmitted HF patients, as per the studies. Low-density lipoprotein (LDL) and triglyceride levels are expected to be higher for readmitted HF patients who did not come in for the initial analysis (refer to Supplementary Table S3).

#### 3.3.4 Fourth Classification

Another set of variables coming out to be essential for readmitted HF patients are diagnostic parameters that include the information about HF patients' LVEF [that came out of the echocardiogram (ECHO) test] and Mitral valve opening values, and whether the HF patient's disease affects one side or both sides of the heart. We can see very conclusively that the Mitral valve opening values for HF patients who underwent re-admission within six months are lower than those who did not get readmitted. LVEF value for readmitted patients is lower than for non-readmitted patients, which looks unusual. Patients with HF affecting both sides of the heart have a higher re-admission percentage than those with only left- or right-sided involvement (refer to Supplementary Table S4).

#### 3.3.5 Fifth classification

The final significant variable identified in the initial analysis was the Discharge Place (destination). This encompasses two factors: the hospital department of discharge and the subsequent location of the HF patient. The study revealed that patients discharged to home, wellness facilities, or locations marked as unknown had a lower readmission rate than other discharge destinations. Moreover, HF patients discharged from the Cardiology department were readmitted more frequently than those discharged from general departments, the Intensive Care Unit (ICU), or other hospital units (refer to Supplementary Table S5).

### 3.4 Feature selection (classification and prioritization)

The selection of the final 44 input variables for the machine learning models involved: Chi-square tests for categorical variables, analysis of variance (ANOVA) for numerical data, and correlation analysis (refer to Supplementary Figures S1, S2, and S6). Because the Charlson Comorbidity Index (CCI) is an additive score reflecting the cumulative burden of comorbidities, it was treated as a continuous numerical variable rather than as a categorical variable in the analysis and in Supplementary Figure S1. In the initial stage, logistic regression, SVM, and random forest classification models were applied to the entire PhysioNet database using 44 variables (cross-validated with the HCUP dataset) for HF patients. This was followed by a refinement process that narrowed to 22 key variables, demonstrating improved model efficiencies, cross-validation scores, sensitivity, specificity, and area under the curve (AUC). The dataset was randomly partitioned into training (80%) and test (20%) subsets, with model development and hyperparameter tuning performed exclusively on the training set. Finally, the research identified seven key variables in the referral process that have nearly the same (or greater) statistical and analytical significance as the 22 variables shortlisted in the second pass of the AI model run. At the time of HF patient discharge, seven key variables of high significance are- Clinical variables, Patient type, Comorbidity, Diagnostic, CCI, Medicine intake, and Discharge details.

### 3.5 Main results: machine learning analysis (classification and prioritization)

The ML models used in this research belong to two model categories: first, interpretable models, such as logistic regression, decision tree, and linear Regression (Bayesian), and second, opaque models, such as SVM and random forest (17, 18). To prioritize HF patients at admission, all ML models were initially tested with 44 variables. Subsequently, through exploratory analysis, basic statistical methods, and the performance of crucial ML models, the number of critical variables was narrowed to 22 in the first and seven in the second phases. The outcomes of the vital models using the 22-variable subset are detailed below:

The outcomes of five machine learning techniques (refer to Table 3) used in the research are below:

- *Logistic model*: model accuracy 69.15%, cross-validation 72.73%, and receiver operating characteristic (ROC) score 64.65% (refer to Figures 2, 3, and Supplementary Figure S7).

**Table 3:** Ranking of HF patients—re-admission.

Patient ID	Chances of re-admission
7,98,704	65.99%
7,38,683	65.36%
8,26,184	64.27%
8,66,996	64.21%
8,60,157	62.12%

The above table shows the ranking of higher-risk HF patients using the logistic regression model.

**Figure 2:**
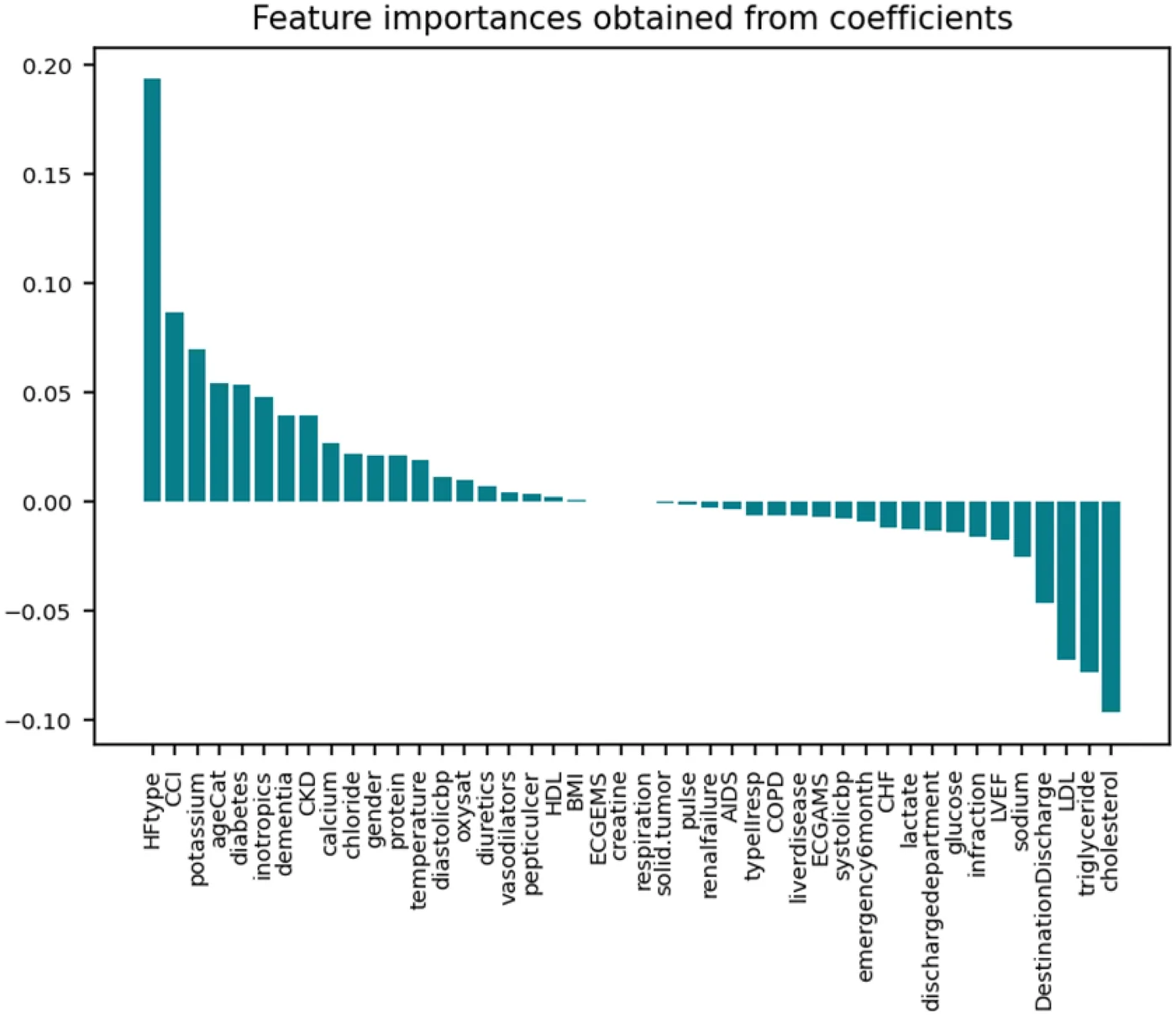
A logistic regression model with shortlisted critical variables for HF patients.

**Figure 3:**
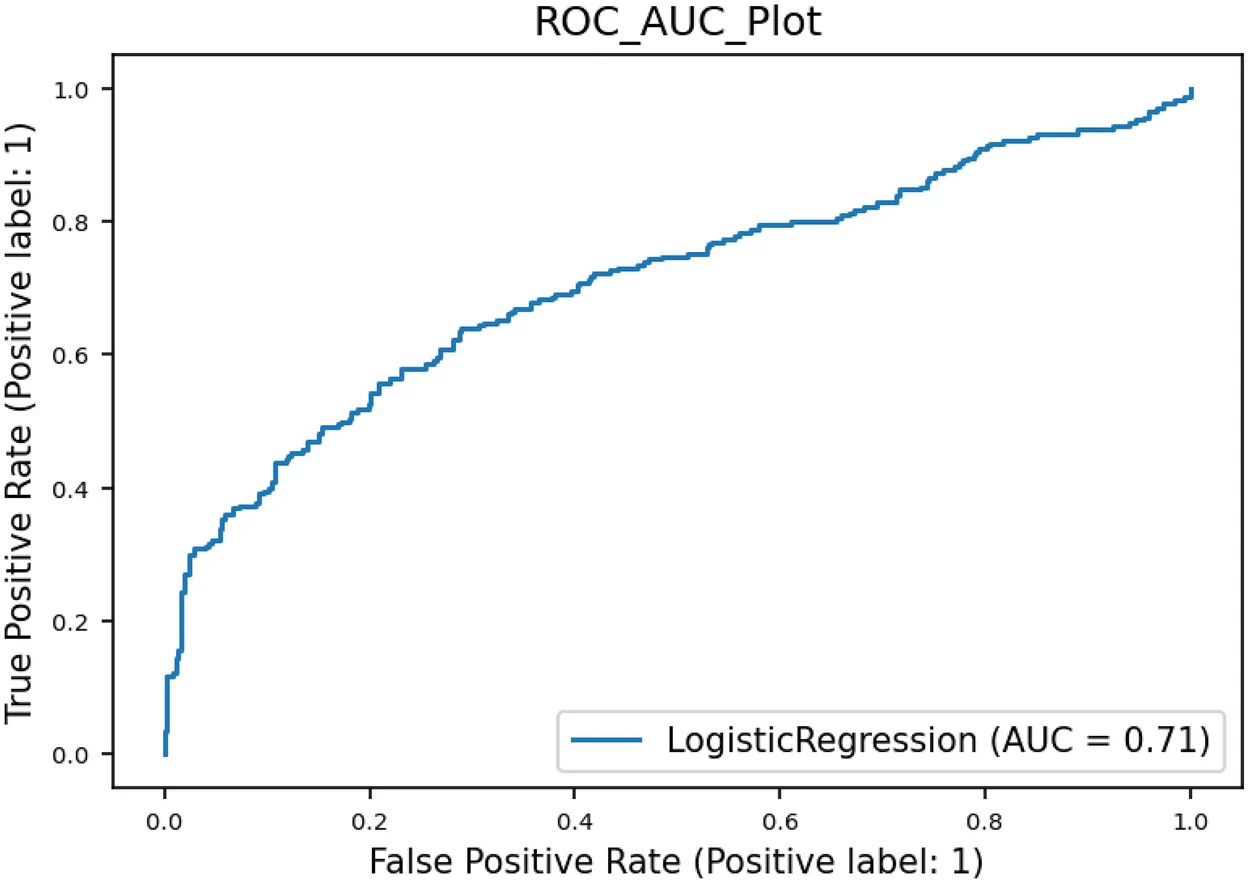
AUC curve of a logistic regression model with shortlisted variables for HF patients.

The logistic model identifies the following health parameters (refer to Figures 2, 3) to be relevant at HF patient referral: These are Diagnostic Variables [Type of HF, 105 Mitral Valve value, Infraction (19) information, and LVEF value], Biochemical/Electrolyte variables (Calcium, Potassium, HDL, LDL, triglyceride), Comorbidities (Diabetes, dementia, CKD, CCI), Patient Type (Gender, Age Cat) and Destination variables (discharge department, destination discharge).

- *Support vector model*: model accuracy 74.30%, cross-validation 74.07%, and ROC score 69.39% (refer to Figure 4 and Supplementary Figure S8).

**Figure 4:**
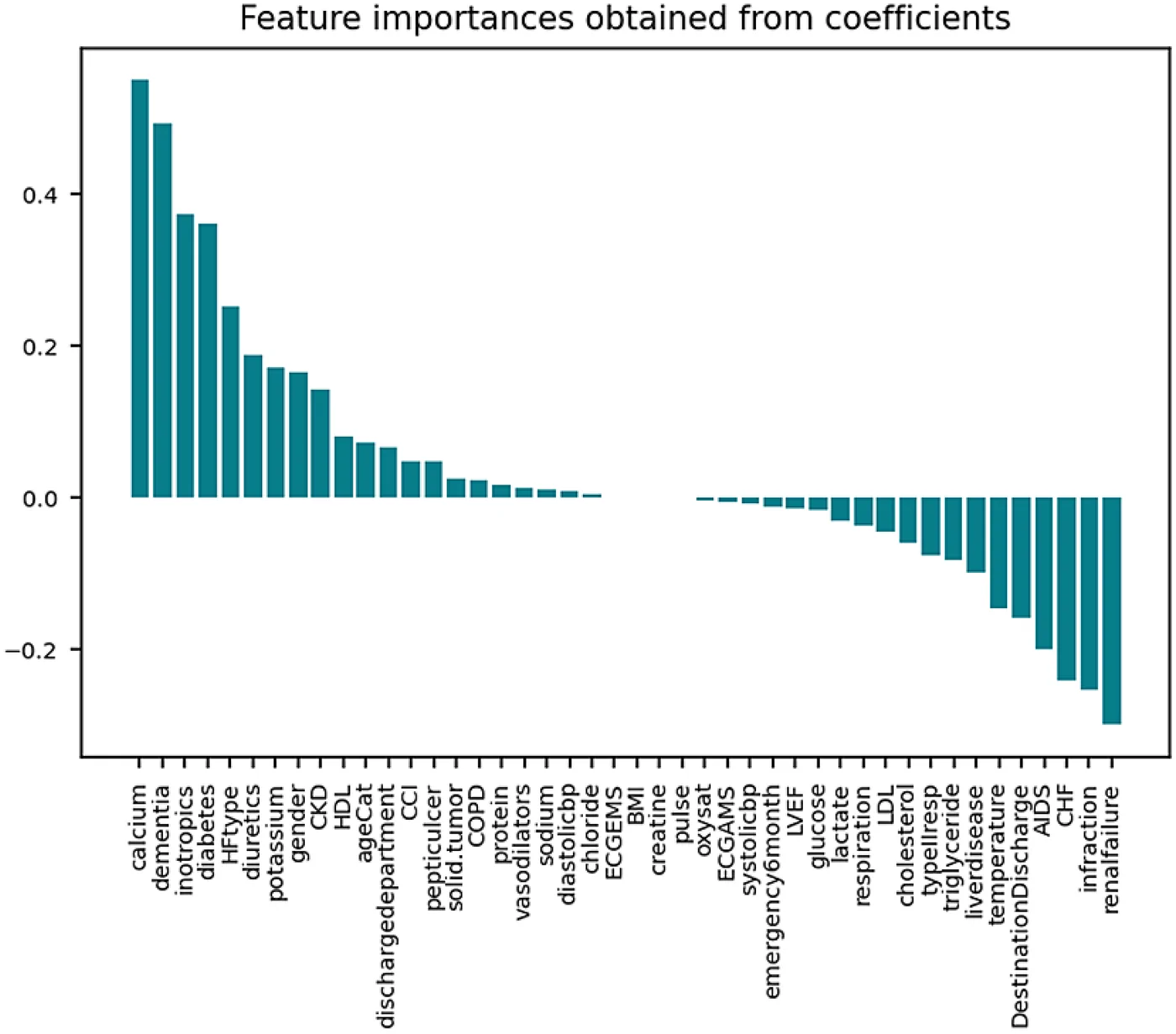
SVM model with shortlisted critical variables for HF patients.

The SVM model identifies the following health parameters (refer to Figure 4 and Figure 5) relevant to HF patient referral: The variable set coming out of the SVM model is composed of Biochemical/Electrolyte variables (Calcium, LDL, HDL), Comorbidities (Diabetes, CCI score, CKD), Patient Type (Gender, Age Category), Discharge Type (destination discharge, discharge department), and Diagnostic variables [LVEF, echocardiogram (ECG), Type of HF, BP].

- *Random Forest model*: The random forest classifier (refer to Supplementary Figures S3, S4, and S9), a supervised learning algorithm used for regression and classification, demonstrated the following performance: 75.46% accuracy, a 78.13% cross-validation score, and an ROC score of 68.83%.

Random forest model identifies the following health parameters to be relevant at HF patient referral: Patient Type (Time to an emergency, Age Category, BMI/Solid Weight), Diagnostic (E/A ratio (ECG), LVEF, Type of HF [electroencephalogram (EEG)], Biochemical/Electrolytes (Triglycerides, LDL, Cholesterol, Potassium, calcium, creatinine), Comorbidities (Dementia, Diabetes, CKD and AIDS), Medicine intake (Vasodilators) and Discharge Type (destination discharge, discharge department). The overall efficiency of the random forest was better than that of logistic and SVM, and raised the bar to improve the model's predictability.

- *K Nearest Neighbor (KNN) model:* By classifying new data points based on their similarity to the closest labeled data points (neighbors) in a training set, this supervised machine learning technique demonstrated a model accuracy of 69.65%, a cross-validation score of 71.34%, and an ROC score of 65.72%

KNN model identifies the following key health parameters to be relevant at HF patient referral: Biochemical/Electrolyte variables (Potassium, Cholesterol, Triglycerides), Comorbidities (Diabetes, CCI score, kidney disease, Dementia), Medicine intake (Inotropic), Discharge Type (discharge department, destination discharge), Patient Type (Age Category, Gender) and Diagnosis [E/A ratio (ECG), LVEF]

- *Advanced ensemble technique*: This unique model is created in the research by combining some of the best classification regression models (20) to implement the machine learning model for predictive modeling more effectively and efficiently (refer to Supplementary Figure S5). The novel hybrid combination of boosting (via XGBoost) with a selection of top classification methods (Decision Tree, Random Forest, Gradient Boosting, and K Neighbors) enhanced the effectiveness of machine learning models, particularly for readmission prediction, where previous model performance was lacking.

Test Accuracy: 74.30%; Cross Validation Score: 76.45%; ROC_AUC Score: 69.64%

This ensemble model identified a similar (to other standard models) set of variable buckets for HF patient referrals, which included Biochemical/Electrolyte variables, Comorbidities, Medicinal Intake, Diagnosis, and Patient and discharge type.

With the improvements achieved through the Hybrid ensemble model, this research has improved the overall odds ratio [Odds ratio depicts the ability of the model to identify the exposure of case patients (HF patients) in general compared to the baseline or control group] of High-Risk HF patients. This step would help risky HF patients to get admission quicker/more easily and address their concerns better than what is currently achieved by the current best-of-the-class classification and regression models, such as XGBoost, Random Forest, and decision trees (18).

- *Linear Regression (Causal Inference/binomial/Bayesian regression):* Five buckets identified by the exploratory analysis during ML modeling were applied in Bayesian regression models (descriptive analytics) to understand the importance of HF patients' discharge/wellness parameters (21) and found to be aligned with earlier analysis. Model accuracy is between 68% and 90% (based on the variables in classification buckets).

Following are the key health parameters found through Bayesian analysis/model: comorbidity parameters (22) [Acquired immunodeficiency syndrome (AIDS), Dementia/medicine, CKD, renal failure, and diabetes], Biochemical/Electrolytes (calcium, cholesterol, HDL, and potassium), Medicinal (Diuretics), discharge destination of HF patients and the department of discharge came out to be the key variables, diagnostic variables, including myocadiac infraction, LVEF, and ECG and patient type data these variables include age category, gender, and BMI. (refer to Supplementary Table S9).

**Figure 5:**
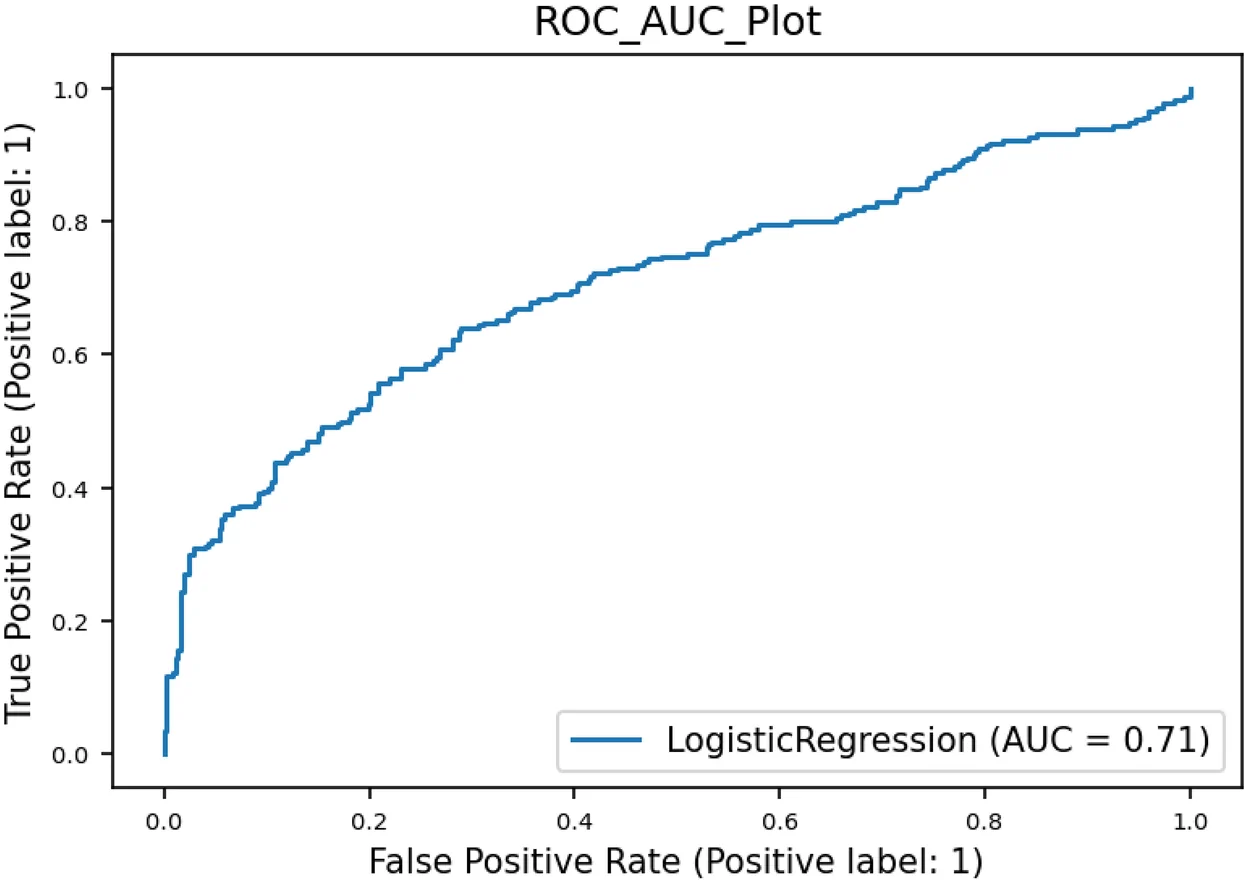
AUC curve of an SVM regression model with shortlisted variables for HF patients.

Table 4 compares seven essential health parameters for HF patients (as identified by ML models) alongside a visual representation of the feature importance of different variables in the various models.

**Table 4:** Mention-comparison table for key machine learning models—re-admission.

Model	Model accuracy	Cross validation	ROC score	Sensitivity	Specificity
Logistic	69.15%	72.73%	64.65%	46%	83.0%
Support Vector	74.30%	74.07%	69.39%	46.5%	85.2%
Random Forest	75.46%	78.13%	68.83%	43.1%	96.78%
KNN	69.65%	71.34%	65.72%	47.8%	78.6%
Advanced Ensemble technique	74.30%	76.45%	69.64%	43.9%	91.4%

The above Table compares the performances of ML models generated in the research.

### 3.6 Model accuracy vs. sensitivity vs. specificity

The three best-performing machine learning (ML) models (based on their outcomes with 25 shortlisted variables) and their critical parameters are detailed below. These models identify essential health parameters for health staff during HF patient admission and rank higher-risk HF patients according to their likelihood of mortality (refer to Table 5).

Model Accuracy (ACC): Model accuracy is significant because it can assess a model's proficiency in processing, comprehending, and predicting HF patients' outcomes (23, 24).

Logistic: 69.15%; SVM: 74.30%; Random Forest: 75.46%; XGBoost: 71.14%; Hybrid (ensemble): 74.30%.

Sensitivity: The sensitivity value quantifies the ML model's capability to correctly identify positive cases, which in this research refers to patients with a higher risk of HF.

Logistic: 46%; SVM: 46.5%; Random Forest: 43.1%; KNN: 47.8%; XGBoost: 50%; Hybrid (ensemble): 43.9%.

Specificity: The specificity value quantifies the ML model's capability to correctly identify negative cases, which in this research refers to HF patients who are not at high risk.

Logistic: 83.0%; SVM: 85.2%; Random Forest: 96.78%; KNN: 78.6%; XGBoost: 84.18%; Hybrid (ensemble): 91.4%.

The models of this study were tuned towards higher specificity to ensure that they reduce the chances of less risky HF patients in higher ML model rankings, thereby managing higher-risk HF patients more effectively.

### 3.7 Verification with HCUP data

The patient prioritization outcomes derived from the PhysioNet dataset were validated against the HCUP MD SID for 2014, a database containing approximately 48,000 HF patient records from Maryland state hospitals, to determine the applicability of the findings in more comprehensive healthcare settings across different geographies and patient populations (refer to Supplementary Figures S6–S9). Five common variable types that were familiar with the PhysioNet HF data were used in the research. These five buckets of the patient's condition at admission (input variables) are Comorbidity, Diagnostic variables, Clinical data, patient type, and discharge location. Key variables from these buckets are Diagnostic variables: (ECHO/LVEF, EKG- Mitral Valve value, EEG, Xray, Cath), Biochemical/Electrolytes variables (Lytes test, COAG) Patient Type (Obesity, Age), Discharge location (ICU, Radiation therapy), Comorbidities (Renal failure/CKD, Chronic Diabetes, Tumor, Ulcer, Chronic Lung).

### 3.8 Ranking of HF patients

The HF patients ranking based on criticality (refer to Table 5) is generated from high to low-risk HF patients. HF patients are categorized based on the chances/probability of getting readmitted to the hospital in the next six months. Patients with a probability from 75% to 100% are considered High-Risk Patients, 50% to 75% are considered Medium-Risk Patients, and less than 50% are Lower-Risk Patients.

**Table 5:** Comparison table for key health parameters for HF patients—re-admissions.

Model	Feature 1	Feature 2	Feature 3	Feature 4	Feature 5	Feature 6	Feature 7
Logistic	Type of HF	Calcium	Discharge Destination	Potassium	Diabetes	Dementia	Myocardial Infraction
Support Vector	Calcium	CCI	Diabetes	Gender	LDL	LVEF	Discharge Destination
Random Forest	Time to Emergency	Type of HF	Triglyceride	ECG	Discharge Destination	Medicine intake	Potassium
KNN	Potassium	Diabetes	Medicine intake	discharge department	Age Category	ECG	Cholesterol
Advanced Ensemble technique	Potassium	Diabetes	Medicine intake	ECG	Discharge Destination	LDL	Age Category

The above Table shows the key features of the ML models applied in the research.

### 3.9 Validation using the CTAS triage model

The critical input variables identified for HF patient readmission using the PhysioNet dataset and verified with the HCUP dataset show similarities to the Canadian Triage and Acuity Scale (CTAS). Specifically, the first key CTAS triage variable, assessing airway breathing, circulation, and disability, aligns with the Patient Type variables in this research. The second CTAS variable, a subjective patient assessment through conversation, corresponds to the comorbidity and medication parameters in this study. The third CTAS variable, identifying key patient complaints, relates to this research's biochemical/electrolyte and diagnostic variables. Finally, the fourth CTAS variable, objective assessment, connects to the HF patient's discharge destination and discharge location (hospital/care unit department).

Beyond this structural mapping, the present findings can be positioned relative to prior triage and readmission-prediction work. The alignment between the model's key variable categories and the four CTAS assessment domains is consistent with the empirical CTAS analysis of Ding et al. (14), which examined how acuity-based prioritization performs in emergency department settings, and with the systematic review of triage performance by Hinson et al. (11), both of which emphasize that a small number of physiologic, symptom, and history-based inputs drive most of the discriminatory value in acuity scoring. In the specific context of HF, the observation that the Charlson Comorbidity Index and its component conditions (notably chronic kidney disease and diabetes) are among the strongest readmission predictors is concordant with Sheng and Xu, who reported that the CCI is independently correlated with six-month readmission in the same PhysioNet cohort, and with earlier electronic health record-deployable risk models for 30-day rehospitalization and mortality in older HF patients reported by Eapen et al. (16). The modest discriminatory performance obtained here (AUC in the 0.65–0.70 range) is also broadly in line with previously published HF readmission models, which have typically reported AUC values in the low-to-mid 0.60 s to low 0.70 s, underscoring that six-month readmission remains difficult to predict from administrative and admission-time clinical data alone. The novel contribution of the present work relative to these reports is therefore not a large gain in raw discrimination but the demonstration that a compact, cross-geography variable set can be applied consistently across two independent health systems (PhysioNet and HCUP) and mapped onto an established triage framework.

## 4 Discussion

This research identifies HF patients with higher chances of hospital re-admission within six months of their previous hospitalization (25) or service related to HF. Not only were high-risk patients with higher chances of re-admission identified, but also variables/parameters were checked for HF patients at the time of discharge (after previous hospital admission) (26) by using a combination of exploratory/confirmatory, machine learning modeling, descriptive analytics, and causal analysis. Five input variables were identified in the PhysioNet dataset and further verified/validated using a different dataset from the US healthcare system (Maryland state data for 2014), containing approximately 48,000 records to cross-check the risk of HF patients getting readmitted after their previous hospital admission/services. Another variable/bucket that came out to be necessary during the analysis of Maryland state data is the consumption of medicines in HF patients (11). Based on crucial input parameters from the six buckets mentioned above, the AI models ranked higher-risk HF patients with a higher chance of getting readmitted (27). These patients should be given special attention, rehabilitation plans, resource allocation, and care to reduce the chances of their future re-admissions in the next six months. These input variables of HF patients help provide effective and focused wellness/rehabilitation at brick-and-mortar facilities or telehealth or virtual care methods (28). HF patients with a re-admission possibility of 75% or above are ranked high-risk re-admission patients. HF patients with a 50% to 75% chance of re-admission are considered a medium risk for re-admission (12), and finally, patients with less than a 50% chance of re-admission are kept at a lower risk (29). Based on risk allocation and possibilities of re-admissions, hospital-related wellness departments can initiate these wellness plans, engage vendors, and government agencies for delivering the right combination of personal visits, online follow-up, and application of tools to determine health conditions. This AI decision support process suggested in the research helps patients, providers, health insurance, and wellness companies to reduce HF patients' risk in a patient-centric approach.

Three critical improvements brought by this research, as compared to the past work, are mentioned below:

*First improvement*- This research overcomes limitations of previous work by providing healthcare staff with a larger set of controllable critical variables for more effective prioritization of HF patients during hospital admissions or readmissions. The variables of interest at the time of admission for HF patients are around 25 [using Patient Prioritization (6, 29)], helping patients seek admission to any hospital/facility to manage their heart condition due to HF or related incidents. Overall, this research not just expanded the base of variables that health staff can control/manage for HF patients at the time of re-admissions for HF conditions but also showcased the outcomes/buckets that can apply to various healthcare settings using observation, clinical, patient type, patient history, simple to complex diagnosis from machines, medicinal intakes, and comorbidities. This work makes this research a good way to apply the findings across the board and easy to implement.
*Second improvement*- A key achievement of this research is its broad applicability across various healthcare systems, from simple to complex due to its ability to leverage seven health parameters with almost similar statistical and analytical significance (Clinical variables, Patient type, Comorbidity, diagnostic, CCI, medicine, and discharge details) in simple health systems to 25 variables in improved/complex health systems. Due to their advanced diagnostic capabilities and knowledgeable staff, complex healthcare systems can evaluate many health parameters for HF patients upon discharge. However, this research, utilizing two datasets (PhysioNet and HCUP), identifies five key variable categories (refer to Table 6) that offer a practical and easily implementable set of health indicators for health staff across different healthcare system complexities and geographical contexts (villages, small towns, or big cities).
*Third improvement*- The generalizability of the HF patient results is a key strength of this research, achieved through a novel six-step process. This process encompassed exploratory analysis, statistical techniques, machine learning modeling, Bayesian regression modeling, validation using a US healthcare system dataset of approximately 48,000 records, and cross-validation with the CTAS patient prioritization model—a level of rigor not consistently seen in past HF research. Consequently, the findings of this study are more effectively applicable across diverse healthcare systems and geographies worldwide.
*Fourth improvement*- Technical and Methodological Improvements for Reliable Outcomes: A key contribution of this research is a new and more effective ensemble technique for predictive analytics. This method provides an alternative to the practical XGBoost approach by innovatively integrating the best aspects of boosting, nearest neighbor, logistic regression, decision tree, and random forest. This hybrid strategy improves machine learning model predictive accuracy and performance by aggregating 157 weak learners—individual models with limited predictive power and prone to overfitting—into a single, robust learning model. Boosting, a machine learning method for error reduction in prediction, is a core element of this approach. The predictive model created in this research is a unique ensemble model to handle various healthcare patient data from geographies and the healthcare system. This newer ensemble model successfully predicted the outcome of HF patients during the patient journey from admission to discharge and very efficiently worked on datasets from two different world geographies and healthcare systems (China and the USA). Overall, this final prediction model generated in this research shows higher model efficiency, specificity, and comparative sensitivity than the single (XGBoost) model, not the ensembled patient prioritization model.

**Table 6:** Comparison table of key variables physioNet vs. HCUP datasets—re-admission.

PhysioNet data	HCUP data (Maryland, USA)
Demographic and Patient Type (Gender, Age Category, Time to Emergency)	Demographic/ Patient Type (Age, Weight Loss)
Comorbidities (Diabetes, CCI score, CKD, dementia, AIDS)	Comorbidities (Depression/Dementia/Stress, Chronic lung, Renal/CKD, Diabetes, Tumor)
Biochemical Variables (Calcium, Potassium, Creatinine, LDL, HDL, triglyceride, cholesterol)	Biochemical Variables (Lytes, Blood report)
Diagnostic variables (LVEF, Mitral Valve value, Type of HF, Blood pressure)	Diagnostic Variables (ECHO, CT scan, x-ray, Cath)
Discharge Type (destination discharge, discharge department)	Discharge Destination (ED, Radiation Therapy)
Medicine Intake (Diuretics, Inotropic, Vasodilators)	Medicine/drug intake

The above Table compares variables generated using PhysioNet and HCUP.

### 4.1 Limitations

Future research should address the following limitations identified in this study. Firstly, the missing data variables within the PhysioNet dataset complicated the imputation process, potentially affecting the accuracy of newly generated data points in reflecting the actual data distribution. Future work could explore more sophisticated imputation techniques or seek datasets with fewer missing values. Secondly, this study's focus on easily recognizable HF cases at admission suggests a need for future research to address the challenges of identifying HF when it overlaps with other cardiac conditions. A related and important limitation is that the primary outcome was defined as all-cause rehospitalization within six months rather than HF-specific rehospitalization, because the PhysioNet source data capture readmission and emergency department return as all-cause events and do not include an adjudicated cause-of-admission field that would reliably separate HF-related from non-HF-related readmissions. This is a meaningful constraint in an older, multimorbid, and frail HF population, in whom the risk of non-cardiovascular hospitalization (for example, infection, falls, or exacerbation of a non-cardiac comorbidity) is also substantially increased; as a result, the model predicts general readmission risk in HF patients rather than recurrent HF events specifically. Consequently, the discussion of comorbidity and wellness-pathway assignment should be read as addressing overall readmission burden in HF patients rather than HF decompensation alone. Future work with linked diagnosis codes at the readmission event should classify readmissions into HF-related and non-HF-related categories and report the breakdown of events used as ground-truth labels, ideally in supplementary material, so that a HF-specific model can be developed and compared against the current all-cause model. Thirdly, the finding that the highest mortality risk does not always correlate with the highest benefit from wellness interventions warrants further investigation to optimize intervention strategies based on individual patient profiles. Fourthly, future studies should aim to obtain data that more accurately reflects the overall burden of HF, as the current research's specific data may underestimate it. Fifthly, the limitation concerns the speed of the models (standard prioritization and referral models) to generate the priority running on the extensive dataset. This research partially handled this problem by creating a Hybrid Ensemble model (with XGBoost) that increased model efficiency and generated faster outcomes. The final limitation is applying a referral prioritization list based on geographic areas, such as age, race, religion, patient comfort, and patient status. All the recommendations for wellness cannot be generalized and need to be considered based on local factors and patient-specific requirements. It should also be noted that, as shown in Table 3, the overall discriminatory performance of the models remains modest, with ROC/AUC scores ranging from 64.65% (logistic regression) to 69.64% (advanced ensemble technique). Models were tuned toward high specificity (up to 96.78% for random forest), while sensitivity remained low across all models (43.1%–47.8%). This means that, while the models are relatively good at correctly ruling out low-risk patients, they miss a substantial proportion of patients who are in fact readmitted within six months. Clinically, this trade-off has direct implications for how the model could be used: as currently tuned, it is better suited to supporting resource allocation for confidently identified high-risk patients than as a stand-alone tool for ruling out readmission risk, and this limitation in sensitivity should be explicitly disclosed to clinical end users before deployment. Future refinement, for example threshold adjustment, cost-sensitive learning, or class-balancing techniques such as SMOTE, should aim to improve sensitivity without unacceptably sacrificing specificity. A further limitation concerns the timing of the predictor data relative to the outcome: because the primary outcome in this study is an all-cause event occurring after discharge, analyses based on data collected at discharge, once the acute pathophysiological state of HF has at least partially resolved, would in principle be preferable. In the present study, however, several predictor variables, namely the baseline clinical characteristics, laboratory findings, and comorbidities, were instead captured on day one of the index admission rather than at discharge (see the Variables subsection), so that they may still reflect the acute presentation of HF rather than the patient's condition at the point of discharge and risk stratification. This timing mismatch could affect the interpretation of the findings in at least two ways: predictors that are elevated or abnormal chiefly because of the acute admission episode may appear more influential in the models than they would if measured at discharge, and the resulting risk scores may be less representative of a patient's actual status when wellness or rehabilitation pathway decisions are made at the time of discharge. Because only the demographic and discharge-related variables (discharge department and discharge destination) were captured at discharge itself, the model's performance and variable importance rankings should be interpreted as characterizing risk based substantially on admission-time status, and future work should evaluate whether re-deriving the clinical and laboratory predictors from discharge-time measurements changes model performance or the ranking of key variables. In summary, the outcome generated from this research can be leveraged in the futuristic healthcare areas beyond health operations by applying AI-based prioritizing and ranking critical HF patients for triage (30), wellness, and emergency treatments (28). For wellness, each patient can be suggested a rehabilitation/wellness path with the proper set of vendors, specialists, tools, or diagnosis scenarios to manage patient risks effectively (31). While our model provides risk stratification for six-month rehospitalization, it does not incorporate a real-time patient prioritization framework across multiple co-existing chronic conditions, an approach that has been proposed in the broader remote-health monitoring literature (32).

## Data Availability

The original contributions presented in the study are included in the article/Supplementary Material, further inquiries can be directed to the corresponding author. The requisite permission was secured to use the PhysioNet and HCUP databases in this study.
