# Supplementary material for: A machine learning model for predicting six-month rehospitalization in heart failure patients with multiple comorbidities
Source: Front Cardiovasc Med. 2026 Sep 18;13:1612592. doi: 10.3389/fcvm.2026.1612592 (PMC13630637; doi:10.3389/fcvm.2026.1612592)
Supplement: Supplementary file 1 [file Datasheet1.docx]

Supplementary Material

# 1 Supplementary Figures and Tables

**Table S1**. Comorbidity Percentage of HF patients- Readmission

| **Key Parameters** | **Readmitted in 6 months** | **Not Readmitted in 6 months** | **% Total Number** |
| --- | --- | --- | --- |
| Liver disease | 36% | 64% | 4% |
| CKD | 46% | 54% | 24% |
| Renal Failure | 29% | 71% | 0.3% |
| Diabetes | 46% | 54% | 23% |
| Dementia | 50% | 50% | 6% |
| COPD | 38% | 62% | 12% |

Note: The Above Table shows the percentage of HF patient readmissions due to comorbidities within six months of the initial hospitalization

**Table S2**. Patient Type data for HF patients- Readmission

| **Key Parameters** | **Readmitted in 6 months** | **Not Readmitted in 6 months** | **% Total Number** |
| --- | --- | --- | --- |
| Age (<60) | 35% | 65% | 9% |
| Age (60<90) | 39% | 61% | 86% |
| Age (90<110) | 42% | 58% | 5.0% |
| BMI (Avg) | 21.68 | 21.85 | NA |
| Gender (Male) | 40% | 60% | 42% |
| Gender (Female) | 38% | 62% | 58% |

Note: The Above Table shows the distribution of readmitted HF patients based on their Age, gender, and BMI

**Table S3**. Biochemical parameters of HF patients- Readmission

| **Key Parameters** | **Readmitted in 6 months** | **Not Readmitted in 6 months** | **Total Cases** |
| --- | --- | --- | --- |
| LDL(Avg) | 1.81 | 1.89 | 1.86 |
| HDL (Avg) | 1.09 | 1.1 | 1.1 |
| Triglyceride (Avg) | 1.13 | 1.17 | 1.16 |
| Calcium (Avg) | 2.3 | 2.28 | 2.29 |
| Potassium (Avg) | 4 | 3.94 | 3.98 |

Note: The Above Table shows some of the critical Biochemical (Electrolytes)parameters that need to be focused on during the analysis of HF patients’ readmission

**Table S4**. Diagnosis Parameters for HF Patients- Readmission

| **Key Parameters** | **Readmitted in 6 months** | **Not Readmitted in 6 months** | **Total Cases** |
| --- | --- | --- | --- |
| LVEF (Avg) | 50.4 | 50.85 | 50.7 |
| Type of HF (Both/Left/Right) | 42.2%/27.2%/35.2% | 57.7%/72.7%/64% | 73.7%/23.7%/2.5% |
| Mitral Valve EMS (Avg) | 3.8 | 5.3 | 4.84 |
| Mitral Valve AMS (Avg) | 3.58 | 4.27 | 4.08 |

Note: The Above Table shows the comparison of diagnostic variables for HF patients, including LVEF and Mitral opening valve values

**Table S5**. Discharge Destination comparison for HF patients- Readmission

| **Key Parameters** | **Readmitted in 6 months** | **Not Readmitted in 6 months** | **Total Cases** |
| --- | --- | --- | --- |
| Discharge Destination (Facility/Home/Unknown) | 41%/39.6%/28.3% | 58.9%/60.3%/71.6% | 21.8%/66.9%/10.5% |
| Place of Discharge (Cardio/General Ward/ICU/other) | 39%/37%/0/37% | 61%/63%/100%/63% | 84.8%/12%/0.5%/2.5% |

Note: The Above Table shows the impact of both the discharge department and the place where the patient is discharged on later readmission

**Figure S1**. Chi-square test for categorical variables- PhysioNet- Referral/Readmission. The chi-square test indicates variables of higher importance for Categorical data.

**Figure S2**. ANOVA test Referral for continuous Variable-PhysioNet. The ANOVA test indicates key variables of higher importance for numerical data.

**Figure S3**. Correlation variables- Admission-PhysioNet. Correlation chat indicates the input variables with a high correlation value with Random Forest.

Note. Accuracy:75.62%; Cross Validation Score:76.66%; ROC_AUC Score:68.96%.

**Figure S4**. Correlation variables- Admission-PhysioNet. AUC curve for Referral- Random Forest.

**Figure S5**. Correlation variables- Admission-PhysioNet. Correlation chat indicates the ensemble model's input variables with a high correlation value.

**Figure S6**. Chi-Square test for state data- MD 2014 (HCUP). Key parameters are comorbidities, such as Liver disease, Diabetes, Renal failure, and Tumor/cancer; Diagnostic variables, such as EEG, EKG, Obesity, and therapy; patient classification variables, such as Chron1 (part of Chronic illness, CCI) and medicine/drug intake.

**Figure S7**. Logistic regression for state data- MD 2014. The above figure shows the importance of HCUP data variables with a logistic regression model.

**Figure S8**. Support Vector Machine regression for state data- MD 2014. The above figure shows the importance of HCUP data variables with the SVM regression model, and it is coming out to be similar to the primary research generated with the PhysioNet dataset.

**Figure S9**. Random Forest for state data- MD 2014. The above figure shows the importance of HCUP data variables with the Random Forest model, and it is coming out to be similar to the primary research generated with the PhysioNet dataset.
